# Optimization of image shoot timing for cerebral veins 3D-digital subtraction angiography by interventional angiography systems

**DOI:** 10.1007/s12194-024-00852-4

**Published:** 2024-10-29

**Authors:** Kazuya Saeki, Takayuki Tamura, Shingo Kouno, Eiji Nishimaru, Masao Kiguchi, Takafumi Mitsuhara, Kazuo Awai

**Affiliations:** 1 Section of Diagnostic Imaging, Department of Clinical Support, Hiroshima University Hospital, 1-2-3 Kasumi, Minami-Ku, Hiroshima, 734-8551 Japan; 2 Department of Clinical Radiology, Faculty of Health Sciences, Hiroshima International University, Higashi-Hiroshima, 739-2695 Japan; 3 Department of Neurosurgery, Graduate School of Biomedical and Health Sciences, Hiroshima University, 1-2-3 Kasumi, Minami-Ku, Hiroshima, 734-8551 Japan; 4 Department of Diagnostic Radiology, Graduate School of Biomedical and Health Sciences, Hiroshima University, 1-2-3 Kasumi, Minami-Ku, Hiroshima, 734-8551 Japan

**Keywords:** 3D-DSA, Cerebral veins, Time-enhancement curve, X-ray delay time setting

## Abstract

3D-digital subtraction angiography (3D-DSA) is essential for understanding the anatomical structure of cerebral veins, crucial in brain tumor surgery. 3D-DSA produces three-dimensional images of veins by adjusting the X-ray delay time after contrast agent injection, but the delineation of veins varies with the delay in X-ray timing. Our study aimed to refine the delay time using time-enhancement curve (TEC) analysis from 2D-DSA conducted before 3D-DSA imaging. We retrospectively reviewed 26 meningioma patients who underwent cerebral angiography from March 2020 to August 2021. Using 2D-DSA, we analyzed arterial and venous TECs to determine the contrast agent’s peak time and estimated the optimal imaging timing. Cases performed near this optimal time were in Group A, and others in Group B, with cerebral venous pixel values compared between them. TEC analysis identified peak times: internal carotid artery: 2.8 ± 0.7 s, middle cerebral artery (M4): 4.1 ± 0.9 s, superior sagittal sinus: 8.3 ± 1.1 s, sigmoid sinus: 9.5 ± 1.3 s, and venous structures near tumors: 7.3 ± 1.0 s. We observed several veins peaking immediately after arterial contrast passage, suggesting the optimal X-ray delay should incorporate the arterial contrast agent’s transit time. Statistical analysis revealed that Group A, with imaging timed to reflect the contrast agent transit time, demonstrated significantly better contrast effects than Group B. The X-ray delay time for 3D-DSA imaging of cerebral veins can be optimized in angiography systems by incorporating the contrast agent transit time, calculated from TEC analysis of cerebral 2D-DSA images.

## Introduction

Understanding the anatomy of cerebral blood vessels is crucial for safely resecting brain tumors. Familiarity with the structures and vascular pathways near the tumor is essential, as cerebral venous anatomy varies significantly, necessitating adjustments in the surgical approach based on venous drainage patterns [1, 2].

Imaging techniques, such as computed tomography (CT) and magnetic resonance imaging (MRI), are traditionally used to understand cerebral vascular anatomy. However, 3D-digital subtraction angiography (3D-DSA) is increasingly employed to provide a more detailed view of vascular anatomy. This technique enables the acquisition of three-dimensional images that can be integrated with bone images for surgical guidance [3–6].

**Fig. 1:**
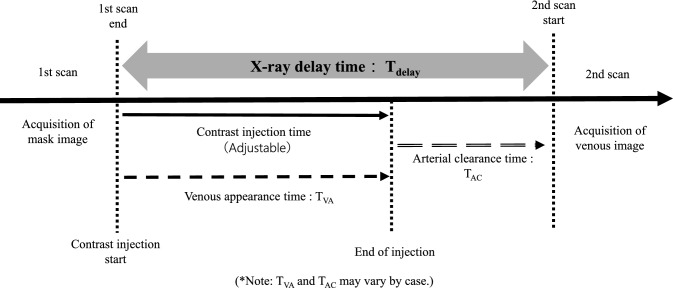
A typical imaging diagram for venous 3D-DSA

In 3D-DSA, the timing of image acquisition after contrast agent injection allows a separate three-dimensional depiction of the arteries or veins. Conventional arterial 3D-DSA involves continuous imaging throughout the entire process of contrast agent injection [7]. In contrast, venous 3D-DSA requires a brief delay after contrast agent injection and before imaging commences, thus necessitating an adjustment of the X-ray delay time [5, 8]. Figure 1 illustrates a typical imaging set up for venous 3D-DSA. The setup ensures that the injected contrast agent reaches the veins from the arteries and that the arterial contrast has cleared before imaging begins. This requires calculating the “Venous Appearance Time (T_VA_)” for the target vein and “Arterial Clearance Time (T_AC_)” from the internal carotid artery to the peripheral part of the middle cerebral artery (M4), based on 2D-DSA images. The sum of these times is set as the X-ray delay time (T_delay_):

1 $$T_{delay} = \, T_{VA} + \, T_{AC}$$

In cerebral venous 3D-DSA, it is essential to continue injecting the contrast agent until the veins are adequately visualized, ensuring that the duration of the contrast injection aligns with the T_VA_. Therefore, the accurate determination of the T_AC_ is critical for setting the precise T_delay_.

Typically, T_delay_ for venous 3D-DSA is determined by referencing 2D-DSA images taken immediately before 3D-DSA imaging. The T_VA_ and T_AC_ are measured through visual inspection and time measurements [8]. Setting the T_AC_ too short leads to mixed images of the arteries and veins, presenting specific challenges. Conversely, overestimating the T_AC_ may result in the contrast agent clearing from the veins during imaging. These issues can ultimately compromise the quality of the imaging outcomes, and we have encountered similar challenges in clinical cases through assessments based on visual measurements.

In this study, we retrospectively examined past cerebral venous 3D-DSA images and aimed to enhance the accuracy of timing determinations by analyzing the time-enhancement curve (TEC) of contrast agents in cerebral blood vessels based on 2D-DSA images. The analysis of TEC enabled the accurate measurement of T_VA_ and T_AC_. We aimed to develop a method to optimize the T_delay_ setting for cerebral vein 3D-DSA imaging. In the following sections, we present our findings and an approach for this optimization.

## Methods

### Equipment

All angiography procedures were performed in an angiographic suite equipped with a flat-panel biplane system (Artis Zee biplane (OR) ICT, Siemens AG, Germany). Image analysis was conducted using the Siemens Syngo Workplace workstation (WS). The TEC of the target blood vessel was acquired using the Syngo i-Flow application integrated into the WS. This application automatically calculates the TEC graph and peak time at a selected point on the DSA image. The contrast agent injector was a Sheen-man ZMC730M (Osaka, Japan), and the contrast agent used was iohexol (300 mgI/ml).

### Participants

In this study, we initially included 28 patients with brain tumors from March 2020 to August 2021, who underwent preoperative cerebral angiography at our institution. All cases included during the period were diagnosed as meningiomas. We excluded two cases where 3D-DSA was performed from the vertebral artery, leaving 26 patients in the study. The clinical characteristics of all cases are shown in Tables 1, 2. The participants had an average age of 58.2 ± 15 years (range 18–79 years), and an average BMI of 23.4 ± 4.1 (range 18.1–33.5), including 9 males and 17 females.

**Table 1:** Clinical characteristics of all cases

Sex	Age	BMI	Tumor site	left and right	Diagnosis
M	59	26.2	Falx cerebri	L	Meningioma
M	71	33.5	Frontal lobe	R	Meningioma
F	52	19.5	Sphenoidal limbus	R	Meningioma
F	55	23.2	Occipital lobe	L	Meningioma
F	71	18.8	Inferior sagittal sinus	L	Meningioma
M	60	23.5	Parietal lobe	R	Meningioma
F	73	21.9	Sphenoidal limbus	R	Meningioma
F	71	25.3	Fornix	R	Meningioma
M	50	21.1	Lateral ventricle	R	Meningioma
F	34	24.6	Olfactory pit	Midline	Meningioma
M	26	18.1	Fornix	L	Meningioma
F	77	19.3	Falx cerebri	L	Meningioma
F	52	32.0	Tubercle of saddle	L	Meningioma
F	71	19.1	Lateral ventricle	R	Meningioma
F	79	23.5	Posterior cranial fossa	L	Meningioma
F	59	18.1	Petrous part	L	Meningioma
M	72	24.5	Petrous part	R	Meningioma
F	56	24.4	Cerebellopontine angle	L	Meningioma
M	49	28.0	Covernous part	L	Meningioma
F	59	24.9	Sphenoidal limbus	L	Meningioma
F	51	19.9	Sphenoidal limbus	R	Meningioma
F	47	24.1	Sphenoidal limbus	L	Meningioma
M	73	26.8	Sphenoidal limbus	R	Meningioma
M	67	26.5	Cerebellopontine angle	L	Meningioma
F	61	31.2	Cerebellar tentorium	R	Meningioma
F	18	20.6	Middle cranial fossa	R	Meningioma

**Table 2:** Peak time of contrast agent concentration for each vessel

	Internal carotid artery	Middle cerebral artery (M4)	
Artery	2.8 ± 0.7 s	4.1 ± 0.9 s	

	Superior sagital sinus	Sigmoid sinus	Near-tumor vein
Vein	8.3 ± 1.1 s	9.5 ± 1.3 s	7.3 ± 1.0 s

For each case, we recorded the vascular imaging data and contrast agent injection parameters of 3D-DSA for retrospective analysis. These parameters included the injection rate, injection time, injected blood vessel, X-ray delay time, and catheter used. The Ethics Review Committee of Hiroshima University approved this study (approval number: E-2629). All imaging was performed for clinical evaluation and not for the purpose of research.

### Imaging protocol and conditions

In all cases, the scan protocol for 2D-DSA involved a tube voltage of 77 kV, a pulse width of 100 ms, a dose of 0.76 mGy/f, and a frame rate of 4 f/s. The contrast agent was diluted 50% with a total volume of 8 ~ 9 ml, manually injected by the physician via the common carotid artery. For 3D-DSA, a 5-s DSA protocol was employed, including a 5-s rotation time, 2 rotations, 200° angular coverage, 133 frames per rotation, a tube voltage of 70 kV, and a dose of 0.36 mGy/f. The reconstruction conditions of 3D-DSA were as follows: kernel type: HU, voxel size: 0.36 mm, matrix size: 512 × 512, image characteristics: normal.

The actual T_delay_ used for 3D-DSA imaging was visually determined using 2D-DSA images in all cases. T_delay_ was set as the time when all veins were judged to be sufficiently visualized on the 2D-DSA images, defined as the actual set T_VA_. Additionally, the time difference between the clearance of contrast from the proximal part of the internal carotid artery and the distal part (M4 segment) after the cessation of contrast injection was defined as the actual set T_AC_. T_delay_ was then calculated using Eq. (1). The contrast agent for 3D-DSA was injected using an automatic contrast injector at an injection rate of 3.5 ml/s(rise time: 0.5 s) with an undiluted contrast agent. The total volume varied with the T_VA_, ranging from approximately 24.5 to 35 ml. The catheters used were either a 4Fr JB2-type or a 4Fr Simmonds-type. The puncture sites were either the brachial artery or the femoral artery, and all procedures were performed under local anesthesia with the patient awake.

### Analysis of TEC and estimation of optimal imaging timing

**Fig. 2:**
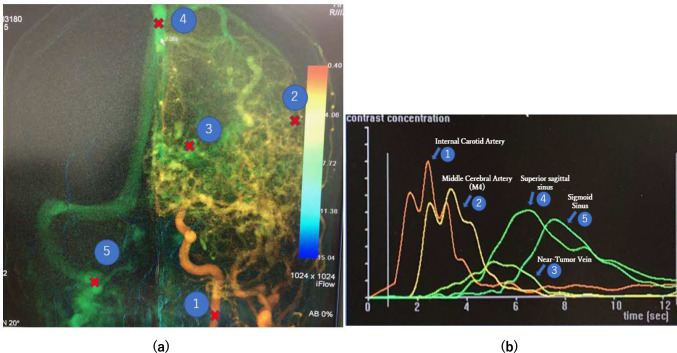
An example of TEC analysis point (**a**) and acquired TEC (**b**). Proximal of Internal carotid artery: ①, Middle cerebral artery: M4 area: ②, Near tumor vein: ③, Superior sagittal sinus: ④, Sigmoid sinus: ⑤

Regions of interests (ROIs) were identified on the target blood vessels within the 2D-DSA images for TEC measurement. The ROIs were designated as the points identified by mouse click. To account for potential errors due to laminar flow, measurements were conducted at three distinct points within each ROI, and the average of these measurements was used for analysis. Primarily, frontal images were utilized for analysis; however, lateral images were used as necessary due to the overlapping of blood vessels. The intracranial internal carotid artery at the lowermost part of the image was selected for TEC measurements as the proximal arterial part, while the distal portion of the middle cerebral artery (M4 segment) was chosen as the peripheral arterial part. For the venous part, the visible veins near the tumor, the superior sagittal sinus, and the sigmoid sinus (dominant side) were selected (Fig. 2a). Using the acquired TEC data (Fig. 2b), we calculated the peak time for each blood vessel, defined as the interval from the onset of contrast injection to the peak of the TEC. For venous vessels, this peak time was used to determine the estimated T_VA_ for each vessel. For arterial vessels, the estimated T_AC_ was determined by calculating the difference in peak times between the proximal arterial part and the distal portion of each artery.

### Image categorization and quantitative evaluation

**Fig. 3:**
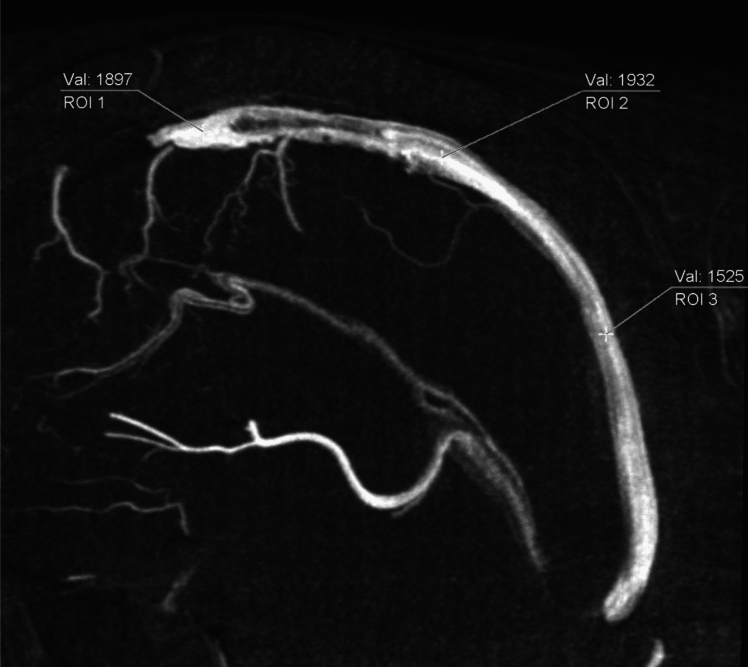
An example of pixel value measurement. (In the case of the superior sagittal sinus.)

In all the cases, 3D-DSA imaging of the cerebral veins yielded three-directional multi-planar reconstruction (MPR) images, encompassing horizontal, coronal, and sagittal sections. The most suitable cross-section was selected for each vessel including superior sagittal sinus, superior cerebral vein, internal cerebral vein, cavernous sinus, sigmoid sinus, and veins near the tumor, and their pixel values were measured. Considering measurement errors due to the overlap of blood vessels, measurements were conducted on 5 mm-thick maximum intensity projection (MIP) images. Measurement points were carefully chosen to avoid areas with reduced signal intensity due to laminar flow effects. The ROI was defined as points identified by mouse click, and the average value of measurements taken at three different locations was recorded as the pixel value (Fig. 3).

The T_AC_, derived from the TEC measured in the sections described in 2–4, was designated as the estimated arterial clearance time (eT_AC_). Conversely, the arterial clearance time visually set with 2D-DSA imaging was termed the actual set arterial clearance time (aT_AC_). A comparison was made between the eT_AC_ and aT_AC_. The actual/estimated time difference ratio (_A/E_TDR) was calculated as follows:

2 $$_{A/E} TDR \, = \, aT_{AC} / \, eT_{AC}$$

For all cases, the _A/E_TDR was calculated. The cases were sorted in ascending order of their _A/E_TDR values. Cases with an _A/E_TDR ≤ 1.2 were classified into Group A, and those with an _A/E_TDR > 1.2 into Group B. The Mann–Whitney U test was used to assess the significance of differences in pixel values between the two groups. Additionally, the _A/E_TDR was varied from 1.2 to 1.5 to similarly classify cases into Groups A and B and evaluate the significance. The significance level was set at P < 0.05 for all tests.

## Result

### Analysis of TEC

The average time taken for the contrast agent concentration to reach its peak value in each blood vessel, as obtained from the TEC is presented in Table 1. In the cerebral arteries, the peak time was 2.8 ± 0.7 s in the internal carotid artery and 4.1 ± 0.9 s in the middle cerebral artery (M4 segment). For the cerebral veins, the peak times were 8.3 ± 1.1 s in the superior sagittal sinus, 9.5 ± 1.3 s in the sigmoid sinus, and 7.3 ± 1.0 s in the veins near the tumor. The earliest peak arrival time was observed in the veins near the tumor, followed by the superior sagittal sinus, with the sigmoid sinus showing the slowest arrival time.

A representative example of the calculated TEC is depicted in Fig. 2b. In the arteries, the peripheral section exhibited a delayed peak arrival time and a lower contrast agent concentration compared to the proximal section, though the concentration curves were similar. In the veins, the order peak arrival was as follows: veins near the tumor, the superior sagittal sinus, and the sigmoid sinus. The peak contrast agent concentration in the veins near the tumor was lower than that in the superior sagittal and sigmoid sinuses. At the peak arrival time in the sigmoid sinus, the contrast agent in the veins near the tumor had nearly cleared. These trends were consistent across all cases.

### Quantitative evaluation of images

**Fig. 4:**
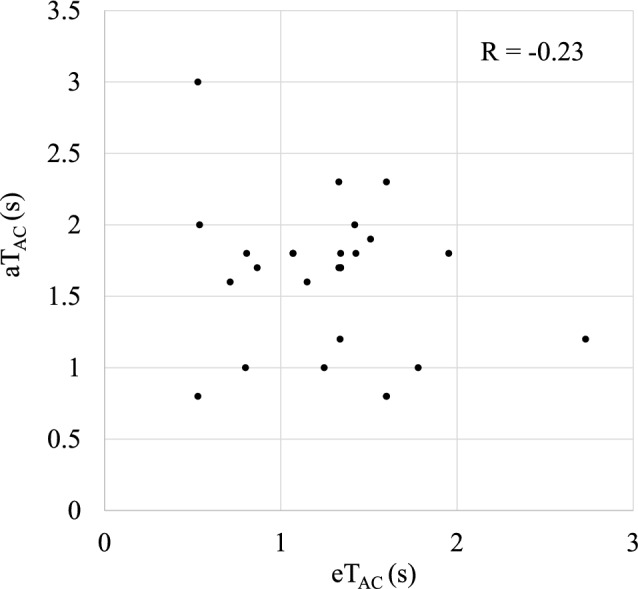
Plot diagram of the relationship between aT_AC_ and eT_AC_ for each case

Table 3 and Fig. 4 show the relationships between the values of eT_AC_ and aT_AC_ for all cases. The mean values of eT_AC_ and aT_AC_ were 1.27 ± 0.49 s and 1.62 ± 0.53 s, respectively. The correlation coefficient (R) was -0.23, suggesting that timing determination based on visual inspection is less accurate compared to using the TEC.

**Table 3:** Actual and estimated arterial clearance time: aT_AC_, eT_AC_ and actual/estimated arterial clearance time ratio: _A/E_TDR for all cases

aT_AC_ (s)	eT_AC_ (s)	_A/E_TDR
1.2	2.73	0.44
0.8	1.60	0.50
0.8	1.60	0.50
1.0	1.78	0.56
1.0	1.25	0.80
1.2	1.34	0.90
1.8	1.95	0.92
1.0	0.80	1.25
1.8	1.43	1.26
1.9	1.51	1.26
1.7	1.34	1.27
1.7	1.34	1.27
1.7	1.33	1.28
1.8	1.34	1.34
1.6	1.15	1.39
2.0	1.42	1.41
2.3	1.60	1.44
0.8	0.53	1.51
1.8	1.07	1.68
1.8	1.07	1.68
2.3	1.33	1.73
1.7	0.87	1.96
1.8	0.81	2.23
1.6	0.71	2.24
2.0	0.54	3.70
3.0	0.53	5.66

The cases were sorted in ascending order of their _A/E_TDR values

**Fig. 5:**
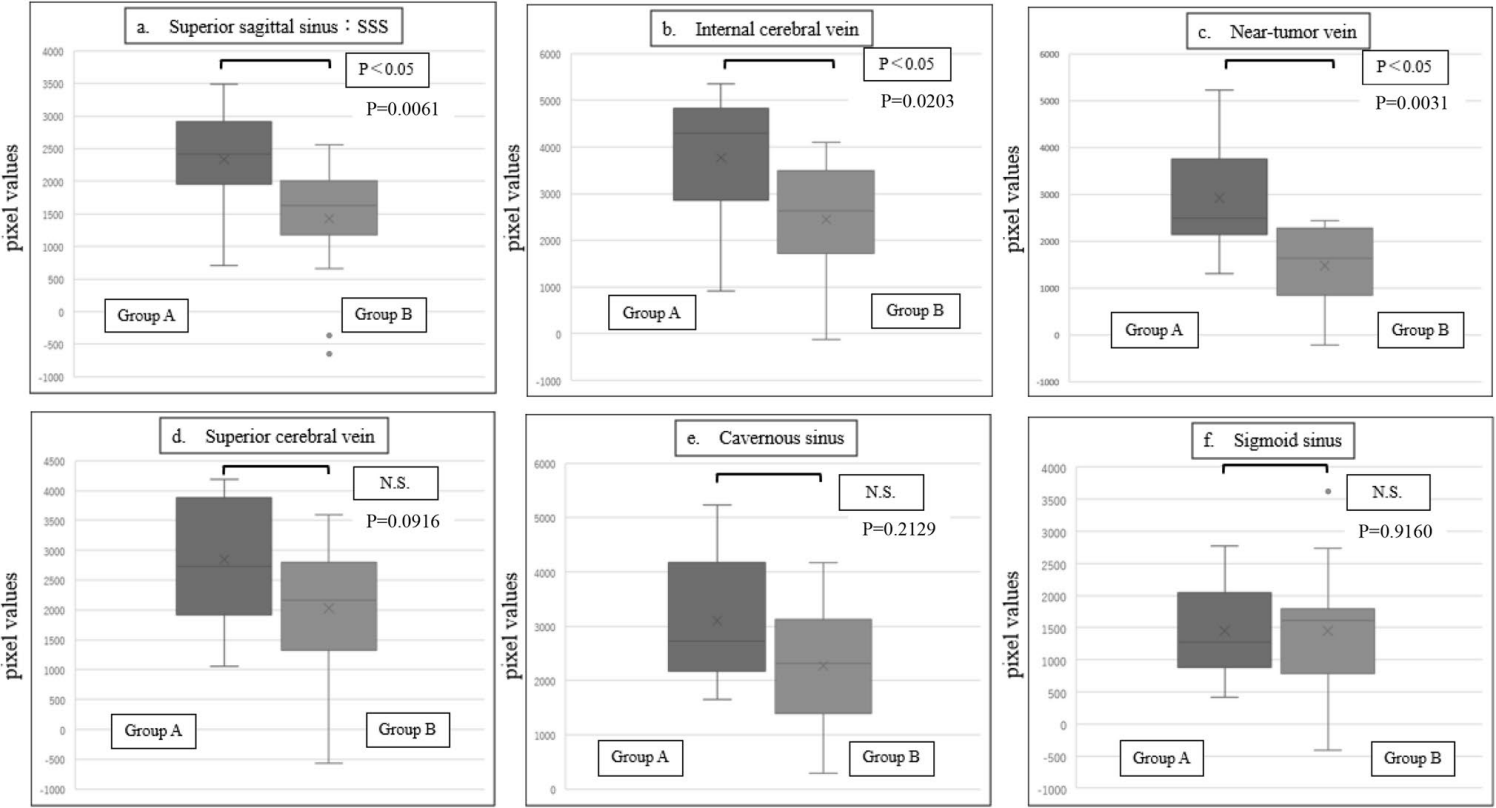
Comparison of pixel values between Group A and Group B for each vessel when _A/E_TDR = 1.2

**Fig. 6:**
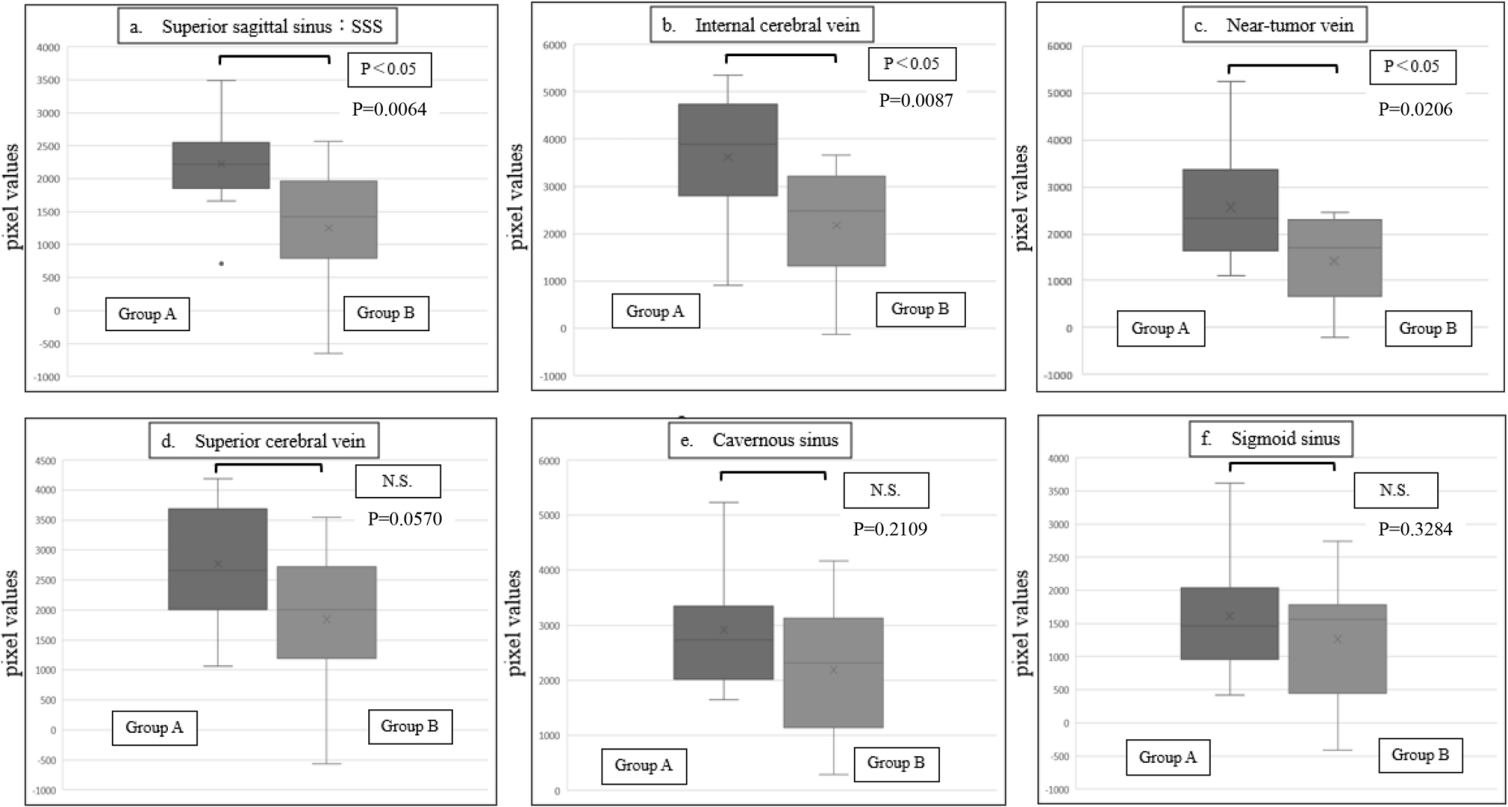
Comparison of pixel values between Group A and Group B for each vessel when _A/E_TDR = 1.3

**Fig. 7:**
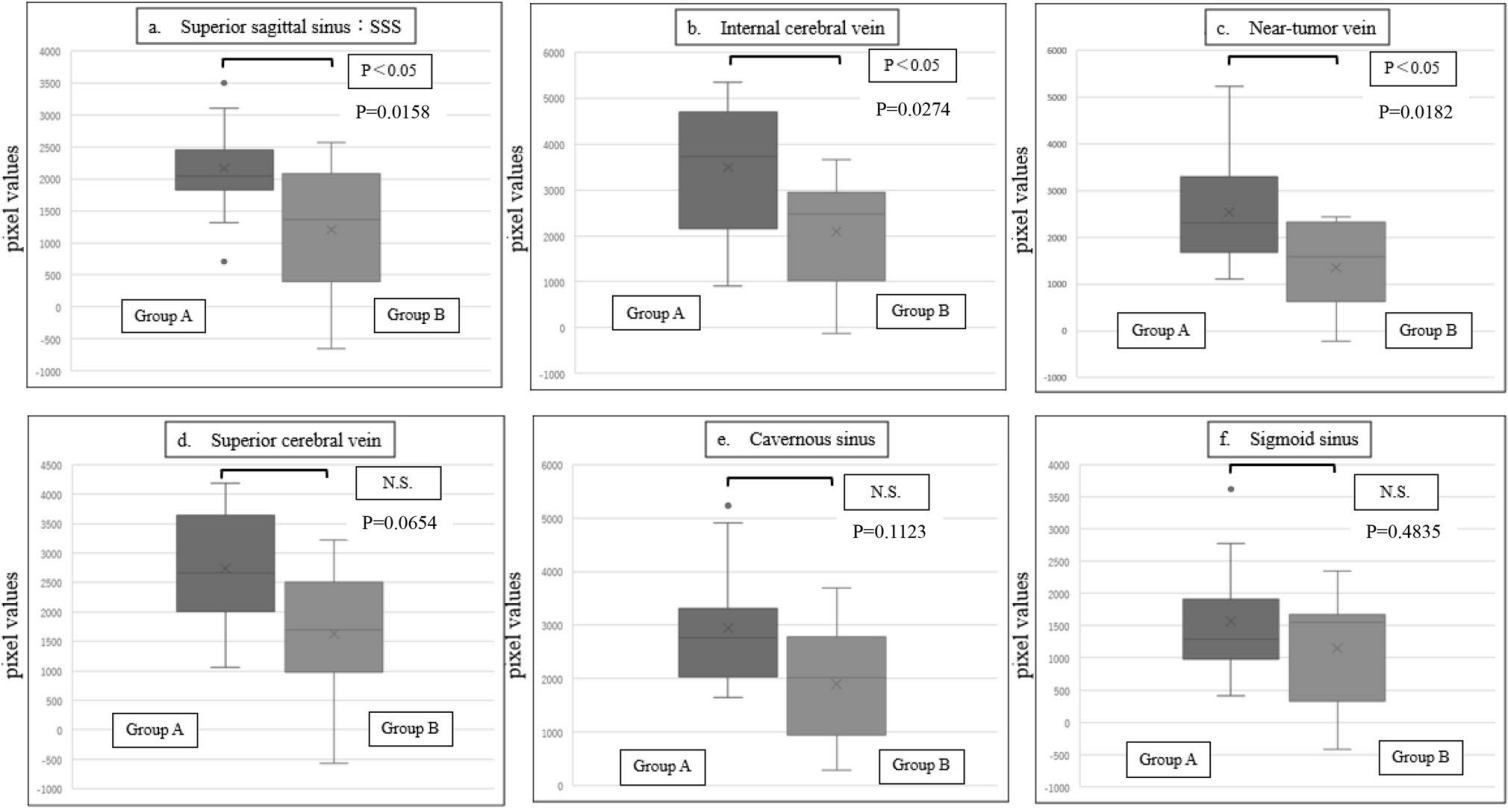
Comparison of pixel values between Group A and Group B for each vessel when _A/E_TDR = 1.4

Figure 5 shows the differences in pixel values for each blood vessel between Groups A and B when _A/E_TDR = 1.2. In Group A, higher pixel values were observed in the superior sagittal sinus (Fig. 5a), internal cerebral vein (Fig. 5b), and veins near the tumor (Fig. 5c). In contrast, no significant differences were found between Groups A and B in the superior cerebral vein (Fig. 5d), cavernous sinus (Fig. 5e), and sigmoid sinus (Fig. 5f). The Mann–Whitney U test confirmed significant differences in the superior sagittal sinus, internal cerebral vein, and veins near the tumor, with P values of 0.006, 0.02, and 0.003, respectively (P < 0.05). As shown in Figs. 6 and 7, increasing _A/E_TDR to 1.3 and 1.4 resulted in gradually larger P values, yet significant differences were still observed. However, when _A/E_TDR was increased to 1.5, no significant differences were found across all venous vessels (P > 0.05), underscoring the sensitivity of the assessment to _A/E_TDR settings.

## Discussion

In this study, focusing on the ‘Venous Appearance Time (T_VA_)’ and the ‘Arterial Clearance Time (T_AC_)’ in the setting of crucial X-ray delay time during cranial venous 3D-DSA imaging, we devised a method to accurately measure the contrast agent arterial transit time using TEC from 2D-DSA. This method was reflected in the X-ray delay time settings, demonstrating its significance.

Visish et al. [9] have reported the utility of intracranial venous 3D-DSA with X-ray delay times set by visual inspection. However, our study’s findings challenge the reliability of this visual method. We found no significant correlation between visually measured aT_AC_ and TEC-calculated eT_AC_ (correlation coefficient R = −0.23), suggested that determining timing based on visual inspection during cerebral vein 3D-DSA imaging does not accurately reflect the optimal timing (Fig. 4). This low correlation coefficient indicates a substantial discrepancy between visual estimation and objective measurement, highlighting the potential inaccuracies in the visual method. Some previous reports [10–12] have pointed out challenges with the visually based inspection methods, even when standardized. Visual assessments are likely varied due to differences in experience and individual subjectivity. Our quantitative analysis supports these previous observations and provides concrete evidence of the limitations of visual assessment. Therefore, for precise venous visualization, referencing TEC is no only advantageous but arguably necessary to achieve consistent and optimal imaging results. Our method provides an objective, quantifiable approach to timing determination, potentially reducing inter-observer variability and improving the overall quality and reliability of cerebral venous 3D-DSA imaging.

TEC analysis for vein near the tumor indicates that early initiation of imaging, following the cessation of contrast agent injection and beyond the influence of arteries, is essential for visualizing tumor-adjacent veins. Kashimoto et al. [13] have proposed a novel protocol for cerebral venous 3D-DSA using low-dose contrast agents, employing TEC analysis of cerebral arteries, cortical veins, and venous sinuses. However, no previous studies have focused on measuring timing with such detail on vessels near tumors, as done in this study, making this method not only novel but also clinically valuable. Furthermore, while providing volume rendering images is useful for understanding the 3D structure of tumors and blood vessels in brain tumor resection surgeries, there is a report indicating that that accurate reproduction of shapes in 3D imaging requires the contrast to be present for more than 80% of the duration of each rotation. [14] Achieving such sufficient contrast effect is crucial, and thus it is important to start imaging only after the contrast agent has adequately filled the veins. Knowing the precise T_VA_ of the target vessel is also essential.

**Fig. 8:**
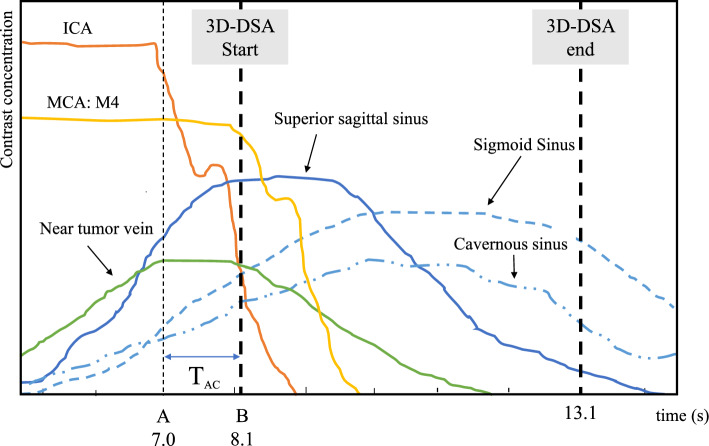
A conceptual model of the TEC under hypothetical 3D-DSA conditions for a case of meningioma. In this case, the contrast agent was injected at a rate of 3.5 ml/s for 7 s (**A**), resulting in a total volume of 24.5 ml of contrast medium used. After the cessation of contrast agent injection, imaging is initiated based on the T_AC_ derived from the TEC obtained from 2D-DSA images, allowing for optimal image acquisition (**B**)

For effective visualization of early enhancing veins during 3D-DSA imaging, it is essential to initiate imaging before the complete clearance of arterial contrast. This study, therefore, focuses on T_AC_, specifically the transit time of the contrast agent from the proximal to peripheral arterial regions. This transit time, calculated by subtracting the peak arrival times between the internal carotid artery and the M4 segment, averaged 1.16 ± 0.2 s for all patients. Figure 8 presents a conceptual model of the TEC under hypothetical 3D-DSA conditions. This model illustrates a scenario where the contrast agent was injected for 7 s until the veins near the tumor were visualized (T_VA_). After the cessation of contrast agent injection, the concentration of the contrast agent in the veins near the tumor and the superior sagittal sinus reached its peak before the arterial contrast was cleared. Therefore, by starting the imaging immediately after the T_AC_ following the cessation of contrast agent injection, the peak contrast in the veins near the tumor and the superior sagittal sinus can be captured for a longer duration. The arterial contrast is cleared immediately after the imaging begins, thus avoiding any interference. Although the peak contrast in other veins occurs slightly later, the contrast concentration remains sufficient during and for some time after the imaging, thereby not significantly affecting the timing of the imaging start. Since the T_AC_ correlates with the intracranial blood flow velocity of each case, it is a crucial factor in determining the optimal T_delay_.

In this study, we placed ROIs on the MCA for TEC analysis due to its accessibility and ease of measurement. Favorable results were obtained even for tumors supplied by other vascular territories, suggesting the clinical viability of this approach. However, if significant differences in clearance times are observed during TEC confirmation, using the TEC of the specific supplying artery would be appropriate. It’s important to note that placing ROIs on peripheral ACA or PCA can be challenging in some cases, which may limit the applicability of this method in certain scenarios.

To validate our theory, we defined the _A/E_TDR and divided the cases into two groups: Group A, where the aT_AC_ used during imaging was close to or shorter than the eT_AC_ calculated from the TEC, and Group B, where it was longer. The threshold was set to _A/E_TDR = 1.2–1.5 for comparison. The superior sagittal sinus, internal cerebral vein, and veins near the tumor, all of which enhanced early, showed significantly higher signal values in Group A compared to Group B. Additionally, as the threshold decreased, the p-values between the groups decreased, suggesting the significance of this method. While no statistically significant difference was observed for the superior cerebral vein, this may indicate that the limited number of cases influenced the results. However, for the cavernous and sigmoid sinuses, where the contrast agent peak appears later and the concentration decreases gradually, leading to no significant difference between Groups A and B.

By using our proposed TEC-based timing measurement method, it is possible to perform imaging at a timing that approaches an _A/E_TDR of 1.0, allowing for detailed venous imaging with a high level of information. Our proposed method for determining the timing of cerebral venous 3D-DSA imaging, based on TEC calculated from 2D-DSA images, can be applied to any angiography system with the same rotation time. Although this study focused solely on the veins of tumors, we believe that this method may potentially be applied to certain cases of arteriovenous malformations (AVMs) and dural arteriovenous fistulas (dAVFs), which can enhance early similar to tumor veins. However, it is important to note that in cases where there is minimal or no time lag between arterial and venous enhancement, such as in high-flow AVMs or dAVFs, this method may not be applicable. The effectiveness of this technique in these conditions would depend on the presence of a discernible time difference between arterial and venous phases. This method might also be useful in the detection of occlusion sites in certain cases of cerebral venous thrombosis, where normal venous drainage patterns are altered. Future studies are needed to validate the applicability and limitations of this method in these diverse pathological conditions.

**Fig. 9:**
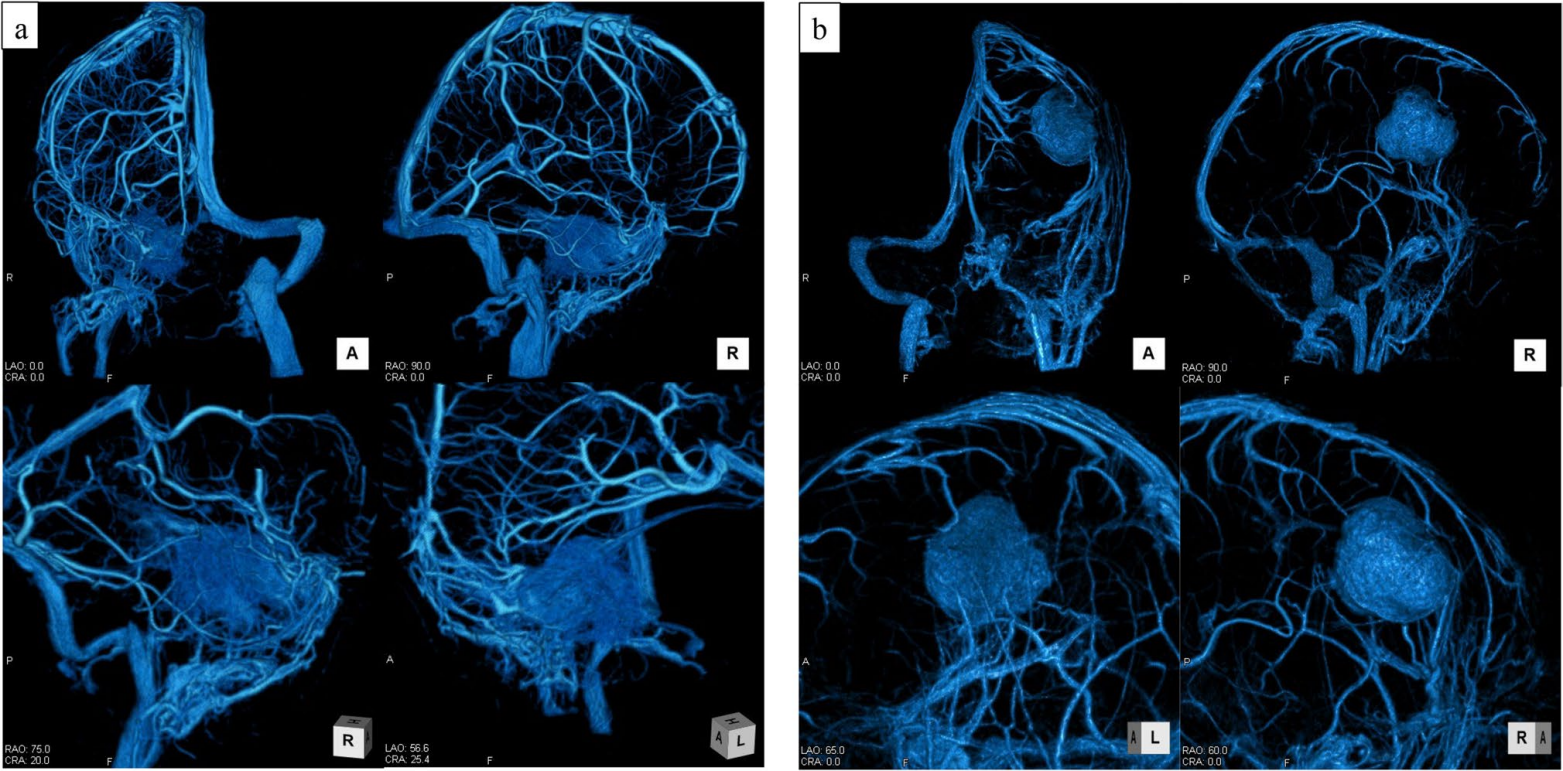
Actual case presentation. In both cases, the contrast agent was injected at 3.5 mL/s for 7.0 s, totaling 24.5 mL. **a** Images obtained from a 72-year-old man with a right petrous part meningioma. (aT_AC_: 1.2 s, eT_AC_: 1.34 s, _A/E_TDR = 0.90) In this case, the area near the tumor to the peripheral vein is clearly depicted without arterial contamination. **b** Images obtained from a 26-year-old man with a left fornix meningioma. (aT_AC_: 2.3 s, eT_AC_: 1.33 s, _A/E_TDR = 1.73) In this case, the veins near the tumor were not visible, and the peripheral veins were poorly delineated

Figure 9 presents actual case images for Groups A and B. In Group A, visualization of peripheral arteries was observed immediately after the initiation of 3D-DSA imaging; however, visualization of the rapidly disappearing veins near the tumor was also confirmed, with no complete disappearance of the contrast agent in the veins during imaging. The 3D images showed no arterial delineation and clear visualization from the entire cerebral vein to the veins near the tumor (Fig. 9a). Conversely, in Group B, there was no initial visualization of peripheral arteries. During imaging, some venous vessels were almost entirely devoid of contrast agents, resulting in insufficient venous visualization in many cases, leading to poor image quality (Fig. 9b).

This study has several limitations. The timing investigation for cerebral venous 3D-DSA imaging was conducted retrospectively using past image data and was solely reliant on TEC analysis. Factors such as patient background (including tumor type, location, size, intracranial pressure status, sex, and age) were not considered. Furthermore, obtaining high-quality venous images prerequisite optimized contrast agent conditions (such as injection rate, volume, catheter type, and vessel positioning). While this study focused on optimizing imaging timing, it is essential in clinical practice that both timing and contrast agent parameters are optimized in tandem. As timing optimization may also lead to the optimization of contrast agent injection volume, future studies should investigate both imaging timing and contrast agent conditions simultaneously. Additionally, this study excluded cases with injections from the vertebral artery. It is necessary to verify whether this method can be applied to cases with injections from the vertebral artery. A more detailed analysis of these factors may enable more precise timing settings in the future.

## Conclusion

The X-ray delay time (T_delay_) setting for cerebral venous 3D-DSA imaging using a vascular imaging system can be optimized by utilizing Vein Appearance Time (T_VA_) and the arterial intravascular contrast agent clearance time (T_AC_), both derived from the TEC analysis of cerebral DSA images. The optimal X-ray delay time was calculated by summing these two times durations.

## Data Availability

The datasets used and/or analyzed during the current study are available from the corresponding author on reasonable request.
